# SAFER MEDICATION MANAGEMENT FOR HOME-DWELLING OLDER ADULTS

**DOI:** 10.1093/geroni/igz038.2785

**Published:** 2019-11-08

**Authors:** Henk Verloo, Armin von Gunten, Boris Wernli, Marie SANTIAGO-DELEFOSSE, Maria Manuela MARTINS, Filipa Pereira

**Affiliations:** 1 University of Applied sciences and Arts Western Switzerland, Switzerland, Switzerland; 2 Service of Old Age Psychiatry, Lausanne University Hospital, Prilly, Vaud, Switzerland; 3 FORS, Swiss Centre of Expertise in the Social Sciences, University of Lausanne, Expert in Database Processing, Lausanne, Vaud, Switzerland; 4 University of Lausanne, Lausanne, Vaud, Switzerland; 5 Institute of Biomedical Sciences Abel Salazar, University of Porto, Porto, Porto, Portugal; 6 University of Applied Sciences and Arts Western Switzerland, Sion, Valais, Switzerland

## Abstract

Taking several medications at the same time can lead to adverse effects and dangerous situations for home-dwelling older adults with chronic conditions. Accurate medication management can be a difficult challenge, especially for people living at home. However, little research has been carried out into the experience of older adults and their informal caregivers with medication management. The aim of the study is, first, to identify factors that can cause undesirable side effects and make taking multiple medications potentially dangerous for home-dwelling older adults. Second, the study will investigate the experiences of this group of patients with medication management. Third, the role of both professional and informal caregivers will be examined. Recommendations will be made on how to improve the safety of medication management for home-dwelling older adults with chronic conditions and should help to prevent the adverse effects and dangerous situations that can lead to hospitalization, institutionalization or premature death

